# Optimization of ultrasound-mediated DNA transfer for bacteria and preservation of frozen competent cells

**DOI:** 10.1128/spectrum.00978-24

**Published:** 2024-11-12

**Authors:** Meng Zhang, Rongkang Tang, Fang-Xia Li, Wen-Yu Jin, Jia-Xin Guo, Lin-Zuo Teng, Guangxun Meng, Philippe J. Sansonetti, Yi-Zhou Gao

**Affiliations:** 1 Shanghai Institute of Immunity and Infection, Chinese Academy of Sciences, Shanghai, China; 2 Suzhou Medical College, Soochow University, Suzhou, Jiangsu, China; 3 Unité de Pathogénie Microbienne Moléculaire, Institute Pasteur, Paris, France; The Hebrew University of Jerusalem, Rehovot, Israel

**Keywords:** ultrasonic plasmid transformation, ultrasonic transformation container, cryopreservation, cryoprotective agents

## Abstract

The transformation of DNA into cells is the basis of molecular biology. Commonly employed techniques include heat shock transformation, electro-transformation, conjugation, transduction, and protoplast fusion. Recently, ultrasonic transformation technology has been developed to transfer DNA into competent cells. The transformation conditions, such as temperature and ultrasonic power, were preliminarily studied. However, this technique has not been widely applied because competent cells must be prepared *de novo*. In this study, various factors, such as ultrasonic frequency and power, were optimized for the ultrasonic transformation of *Escherichia coli*. The study found that the optimal conditions for ultrasonic transformation with a defined ultrasonic transformation vial were a frequency of 28 kHz and a power of 80 W. Meanwhile, this research demonstrated that combining the 42°C heat shock conditions with ultrasonic transformation is the most efficient method compared to using only heat shock. Furthermore, the cryoprotective agent ratio for ultrasonic competent cells was investigated and optimized. These findings provide new insights into enhancing transformation efficiency and lay a foundation for the broader application of ultrasonic transformation.

**IMPORTANCE:**

Plasmid transformation is widely applicable in gene expression and modification. As an efficient, non-invasive, and gentle method of transformation, ultrasonic transformation provides a novel approach for strain modification. This research presents new strategies for enhancing transformation efficiency and lays the groundwork for expanding the utilization of ultrasonic transformation.

## INTRODUCTION

Biotransformation techniques are widely used in various biological fields, especially microbiology, where the transfer of genetic material from bacteria has been intensively studied (1). Genetically modifying microbes in the environment and applying them in agriculture, medicine, and biotechnology are now crucial research ideas. External stimuli, including physical (electroporation and microinjection), chemical (chemical transfer method), and biological (conjugation and transduction) stimuli, are usually required to transfer exogenous DNA into bacteria due to the weak transformation ability of most microorganisms in the environment (2, 3).

The commonly used methods in experiments are heat shock transformation, electroporation (4), and conjugation (5). The heat shock transformation method promotes the absorption of DNA complexes into cells by treating competent cells at a specific temperature (42°C) for a short time (6, 7); also, the addition of calcium ions changes the permeability of the cell wall more favorably to manipulate the bacteria. Electroporation effectively transferred the plasmid into cells using high-voltage electric shock (8). However, the low ionic levels of medium- and high-voltage conditions required in operation may lead to significant cell death (9). Conjugation is a specific process for transferring DNA, which requires specific donor bacteria and plasmids, making the process complex (10–12). With the evolution of biotransformation methods, a new technique has recently been developed to deliver exogenous DNA or other molecules into cells via low-frequency ultrasound. Ultrasound-mediated methods can successfully transform plasmids into some Gram-negative bacteria (13, 14), preliminarily demonstrating the feasibility of ultrasound-mediated transformation of exogenous DNA in prokaryotes.

Low-frequency ultrasound produces microbubbles and cavitation in a liquid to enclose macromolecules, such as exogenous DNA (15). Many unstable microbubbles may rupture under the continuous action of ultrasound. Meanwhile, the energy released by the bursting of microbubbles creates many reversible nanochannels in the competent cell wall and the formation of microflows outside the cell (16, 17), thus facilitating the entry of exogenous DNA into the cell. Consequently, low-frequency ultrasonic transformation (UT) has many advantages over other transformation methods, such as less stringent requirements for experimental conditions, simple and safe operation, and common and economical equipment (18). Additionally, low-frequency ultrasound transformation is less invasive because exogenous DNA is delivered into the cell via sonoporation without directly damaging the cell (9). The existing research (14) used 48-kHz ultrasound for exogenous DNA transformation of *Escherichia coli* HB101. However, its transformation efficiency at optimal transformation conditions was lower than that of the chemical or electro-transformation methods. Therefore, the physical conditions and parameters of the ultrasonic transformation method must be optimized. The laboratory typically utilizes compact ultrasonic instruments with a power range of 80–300 W and a frequency range of 28 or 40 kHz (19). A flat-bottomed glass vial was generally chosen as the container for ultrasonic treatment (13), and the vial was placed in the center of the ultrasonic instrument. However, the detailed parameter for glass vials remains undescribed.

Parallel to the transformation process, the state of competent cells is also a significant determinant of transformation efficiency. Cryoprotective agents (CPAs) are employed to maintain the viability of competent cells during storage in a −80°C refrigerator. Considering the time-consuming preparation of chemically competent cells, it is necessary to explore more effective CPAs. The majority of commercially available *E. coli* competent cells use glycerol (GLY) as a cryoprotective agent (20). GLY can protect cells during cryopreservation by reducing ice crystal formation and osmotic pressure (21). Hollander and Nell (22) discovered that 15% GLY protected *E. coli*, *Shigella flexneri,* and *Haemophilus influenzae* from freeze-thaw damage. Dimethyl sulfoxide (DMSO), an alternative antifreeze, is most commonly used for mammalian cell cryopreservation but is toxic to a certain extent. The research (23) used 1.5 M DMSO to slowly freeze the follicles of canine ovarian tissue, which did not affect their structural integrity and vitality after thawing. DMSO, a CPA component, has also been used for bacterial cryopreservation (24). Furthermore, the penetration rate of antifreeze affects cell survival during cryopreservation. Ethylene glycol (EG) has a lower molecular weight and faster penetration rate, which affords it the potential to protect cells during cryopreservation. A previous study used CPA with 2%–40% EG concentration to freeze certain groups of microorganisms (25).

In this research, various factors that affect the efficiency of ultrasonic transformation, such as frequency, power, the material of the container, and frozen storage of the competent cells, were adjusted to meet the optimal conditions. Furthermore, the optimal composition and ratio of CPAs for the ultrasonic transformation of competent cells have also been reported. This study provides an optimal condition for the method of exogenous DNA transformation using a low-frequency ultrasonic technique and provides a new idea for transformation.

## MATERIALS AND METHODS

### Bacteria and plasmids

*E. coli* DH5α (Trans5α Chemically Competent Cell from TransGen Biotech, CD201) and *E. coli* Top10 (Top10 Competent Cells from Sangon Biotech, B528412) were used as the plasmid recipient. The plasmids used in this study are summarized in Table 1. Among them, pUC18 (26) was preserved in the laboratory, pMG36e (27, 28) was purchased from HonorGene, pBAV1K-T5-*lux* (29), and pCM-*dmpR-lux* (30) were modified in the laboratory. Bacteria were grown in Luria-Bertani (LB) medium at 37°C. Agar was added at a final concentration of 15 g/L for the solid media.

**TABLE 1:** The plasmids mentioned in the determination of ultrasonic transformation

Plasmid	Size (kb)	Characteristic
pUC18	2.7	Amp^r^ (working concentration: 100 µg/mL)
pMG36e	3.6	Ery^r^ (working concentration: 5 µg/mL)
pBAV1K-T5-*lux*	8.6	Kan^r^ (working concentration: 50 µg/mL)
pCM-*dmpR-lux*	18.7	Tc^r^ (working concentration: 200 µg/mL)

### Preparation of competent cells

Commercially competent DH5α and Top 10 cells were streaked onto LB agar plates and cultured for 12 h. A single colony was selected and inoculated into 5 mL liquid LB medium at 200 rpm and 37°C overnight. Briefly, 1 mL of the bacterial solution was inoculated into 100 mL of liquid LB medium and cultured under the same conditions. When the OD_600_ reached 0.4–0.6, the cells were centrifuged at 4,000 rpm and 4°C for 10 min to collect the cell precipitate. Then, the cells were washed twice with 10 mL of precooled calcium chloride (CaCl_2_) solution (100 mM) and suspended with 1 mL of the same solution before being divided into appropriate containers.

### Ultrasound equipment

Ultrasonic cleaners A (Xiaomei Ultrasonic Instrument Co., Ltd, Kunshan, China): 28 kHz, its power could be adjusted from 20 to 200 W. Ultrasonic cleaners B (Fuyang Technology Group Co., Ltd., Shenzhen, China): 40 kHz, the power was 80 W. The following three sample vials were used as experimental containers: a 3-mL ultrasonic transformation vial, a 2-mL autosampler vial (Agilent: 5182-0553), and a 1.5-mL Eppendorf (EP) tube.

### Ultrasonic transformation system

Plasmids were added to 100 µL of DH5α or Top10 competent cell suspension and ultrasonically treated after a 0.5 h ice bath. The plasmid concentration in the transformation system was ensured to be the same as 0.9 ng/µL. The ultrasonic treatment time was 25 s. During ultrasonic treatment, the sample vial was placed in the center of water-filled ultrasonic cleaners A and B. After ultrasound treatment, the bacterial solution was incubated at 37°C for 1 h and diluted. Moreover, the 100-µL diluent was coated on an LB plate (containing antibiotics of the type listed in Table 1) and incubated at 37°C overnight. Each set of experiments had three replicates, and the transformation efficiency was calculated as colony-forming units (CFUs) per 1 µg plasmid. The CFU number was calculated through plate counting according to the dilution level. Each single colony on the plate was calculated as one CFU.

### Different ultrasonic frequencies and power on transformation determination

The experiment was designed to explore the effect of low-frequency ultrasonic treatment on transformation efficiency under the following conditions: the power of both instruments was 80 W, the temperature was 35°C, the ultrasonic treatment time was 25 s, and the plasmid pUC18 concentration in the transformation system was 0.9 ng/µL.

The effect of the ultrasonic power was investigated by setting the ultrasonic frequency to 28 kHz, and 40 W (31) and 80 W power (selected by pre-experiment, and data are presented in Fig. S1) was used to optimize the transformation conditions. In addition, the experiment was designed at a temperature of 35°C, ultrasonic treatment time of 25 s, and plasmid pUC18 concentration in the transformation system of 0.9 ng/µL.

### Effects of different vials on the transformation efficiency

**Fig 1:**
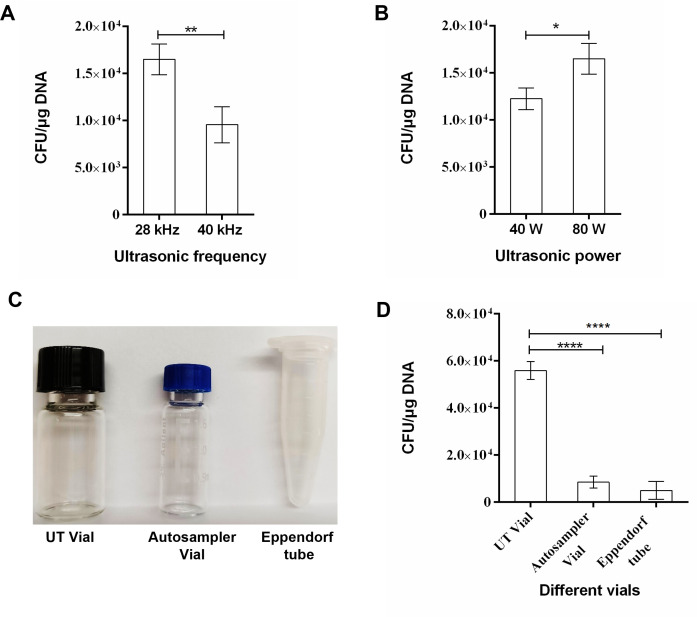
The influence of ultrasonic factors and vials on the transformation efficiency. The host bacteria were DH5α, and the transformed plasmids were pUC18. (**A**) Transformation efficiency at 28 and 40 kHz. (**B**) Transformation efficiency at 40 and 80 W. *0.01 < *P* < 0.05; **0.001 < *P* < 0.01. (**C**) Three experimental containers of different materials. From left to right, the sample vials are a 3-mL UT vial, a 2-mL autosampler vial, and a 1.5-mL EP tube. (**D**) The transformation efficiency after using three vials. ****P < 0.0001.

The ultrasonic wave penetration of vials depends on the material used. UT vials, autosampler vials, and EP tubes (Fig. 1C) were used as experimental containers for ultrasonic transformation treatment under the same conditions (28 kHz, 80 W, 35°C, 25 s, and 100 µL DH5α competent cells), and the concentration of plasmid pUC18 in the transformation system was 0.9 ng/µL. Three sample vials were placed at the center of the ultrasonic cleaning machine during treatment.

Furthermore, the thickness of the vial wall, center of the vial bottom, edge of the vial bottom, and diameter of the vial bottom of different containers were measured using a digital display thickness meter (Bang Te Precision Measurement Tools Co. Ltd, Henan, China) (Table 2).

**TABLE 2:** Technical parameters of ultrasonic transformation vessel^*a*^

	Wall thickness	Bottom thickness		Diameter	Material
		Center	Edge		
UT vial	1.03	0.77	0.91	15.17	Borosilicate glass
Autosampler vial	0.77	0.86	0.59	13.67	Borosilicate glass
EP tube	0.9	1.08	ND	5.13	Polypropylene

^*a*^ ND, Not detected. The unit of data in the table is millimeters.

### Effect of ultrasonic treatment on heat shock transformation efficiency

Since the conventional heat shock and ultrasonic-transformed competent cells were in an environment with a 100 mM CaCl_2_ solution, the synergistic effect of heat shock and ultrasonic treatment on plasmid transformation was attempted in this study to obtain higher efficiency. After 30 min in an ice bath, three groups of experiments were designed: group A (Heat shock) was treated at 42°C for 30 s; group B (ultrasonic transformation) was treated at 25°C, 28 kHz, and 80 W for 30 s; and group C (UT + Heat shock) was treated at 42°C, 28 kHz, and 80 W for 30 s. The common conditions of the above three groups of experiments were 100 µL of DH5α or Top10 competent cells, and the plasmid pUC18 concentration in the transformation system was 0.9 ng/µL. Additionally, plasmids pMG36e, pBAV1K-T5-*lux* and pCM-*dmpR-lux* were used to explore the application range of ultrasonic transformation at the plasmid level.

### Exploring the ultrasonic transformation efficiency and cytotoxicity of competent cells prepared with cryopreservation agent

The antifreeze agents included DMSO, EG, and GLY. Different antifreeze and CaCl_2_ solution (100 mM) ratios were used to prepare CPA to protect the competent cells during cryopreservation (Table 3), and the transformation efficiency after cryopreservation was measured. In the study of cryopreservation conditions, an extra washing process of CPAs was added after the CaCl_2_ solution was washed twice to ensure that the prepared CPAs were in the final freeze-storage environment of the competent cells. Thus, the influence of residual CaCl_2_ solution on the ratio of the final cryogenic protective solution was avoided. The transformation efficiency of competent cells stored in different CPAs was measured using ultrasonic transformation before and after cryopreservation. The transformation conditions were 100 µL of DH5α competent cells, plasmid pUC18 concentration in the transformation system of 0.9 ng/µL, and ultrasonic treatment for 10 s (28 kHz, 80 W, and 25°C).

**TABLE 3:** The ratio of antifreeze-CaCl_2_ solution in cryoprotective agent^*a*^

Antifreeze	The ratio of antifreeze CaCl_2_ solution			
DMSO	1:99	5:95	10:90	20:80
EG	20:80	40:60	50:50	68:32
GLY	10:90	15:85	20:80	30:70

^*a*^ The data in the table are the volume ratio of antifreeze to CaCl_2_ solution.

The toxicity of CPAs on DH5α competent cells was tested to explore the effect of antifreeze treatment on the survival of competent cells. After washing two times, the cells were suspended in 13 mL of CaCl_2_ solution and evenly divided into 13 parts. The cells were rewashed with CPA and suspended in 100 µL of CPA. Notably, the control group was only treated with CaCl_2_ solution from beginning to end. These 13 groups of cells were reasonably diluted and coated on an LB medium for culture, allowing the number of viable bacteria to be counted and calculated clearly.

### Statistical analysis

The transformation efficiencies expressed as a function of several experimental conditions were analyzed using ANOVA or *t*-tests, and this value was considered statistically significant when *P* < 0.05. Each data set was calculated from three replicates. The data were analyzed by GraphPad Prism 6 software (http://www.graphpad.com).

## RESULTS

### The plasmids successfully entered the cells after ultrasonic transformation

Transformed host bacteria were grown in an LB plate (5) (containing antibiotics of the type listed in Table 1) (Fig. S2) and counted after gradient dilution to facilitate the calculation of ultrasonic transformation efficiency. Accordingly, four kinds of plasmids were successfully transformed into the host strain DH5α via ultrasonic transformation.

### Exploring the optimal conditions of ultrasonic frequency and power

A comparison of the influence of ultrasonic frequency on the transformation efficiency (Fig. 1A) indicated that 28 kHz had a higher transformation efficiency than 40 kHz at the same power (80 W). A *t*-test analysis revealed a significant difference (*P* < 0.01).

The power of ultrasound equipment commonly used in laboratories is 80 W, while 40 W is typically used for ultrasonic transformation (31). The efficiency of ultrasonic transformation (Fig. 1B) was explored at 28 kHz with powers of 40 and 80 W. The *t*-test analysis depicted that the transformation efficiency was significantly higher when the power was 80 W (*P* < 0.05).

The entry of exogenous DNA into host cells is mainly dependent on the cavitation formed on the cell surface by ultrasound (32). Ultrasound of low frequency and power acts on the host cells to reversibly change the permeability of the cell membrane, so it causes minimal damage to the cells and can maintain the integrity of the plasmid and the activity of the host cells (33). According to the above data, the optimal ultrasound power was 80 W at the optimum frequency of 28 kHz.

### Effect of experiment container on transformation efficiency

The UT vial group demonstrated the highest transformation efficiency (Fig. 1D), significantly different from the Autosampler and EP tube groups. Although the best container identified in this study is the ordered UT vial, it is also a reasonable choice, given the standardization and accessibility of Agilent 2 mL autosampler vials.

The energy of ultrasonic waves acting on the transformation system is related to the wave propagation medium, material absorption, and other factors. Previous research has proven that plastic absorbs ultrasonic energy and that the smooth round bottom reflects ultrasonic energy, but no specific data have been provided (13). When the ultrasonic wave passes through the plastic material, it will produce elastic energy, internal consumption energy, and other ultrasonic energy consumption. The UT and autosampler vials were made of borosilicate glass, characterized by low metal content, to prevent sample deterioration or leaching (34, 35). Simultaneously, because borosilicate glass contains higher SiO_2_ and B_2_O_3_, it has a lower coefficient of thermal expansion than ordinary glass, higher thermal impact strength, and higher surface hardness (36, 37). By comparing the parameters of UT and autosampler vials, it is found that the bottom diameter of the UT vial is larger, and the center wall of the bottom is thinner. This may be beneficial for ultrasonic waves to pass through the medium of the bottle and act on the transformation system.

### Ultrasound enhances the efficiency of heat shock transformation

**Fig 2:**
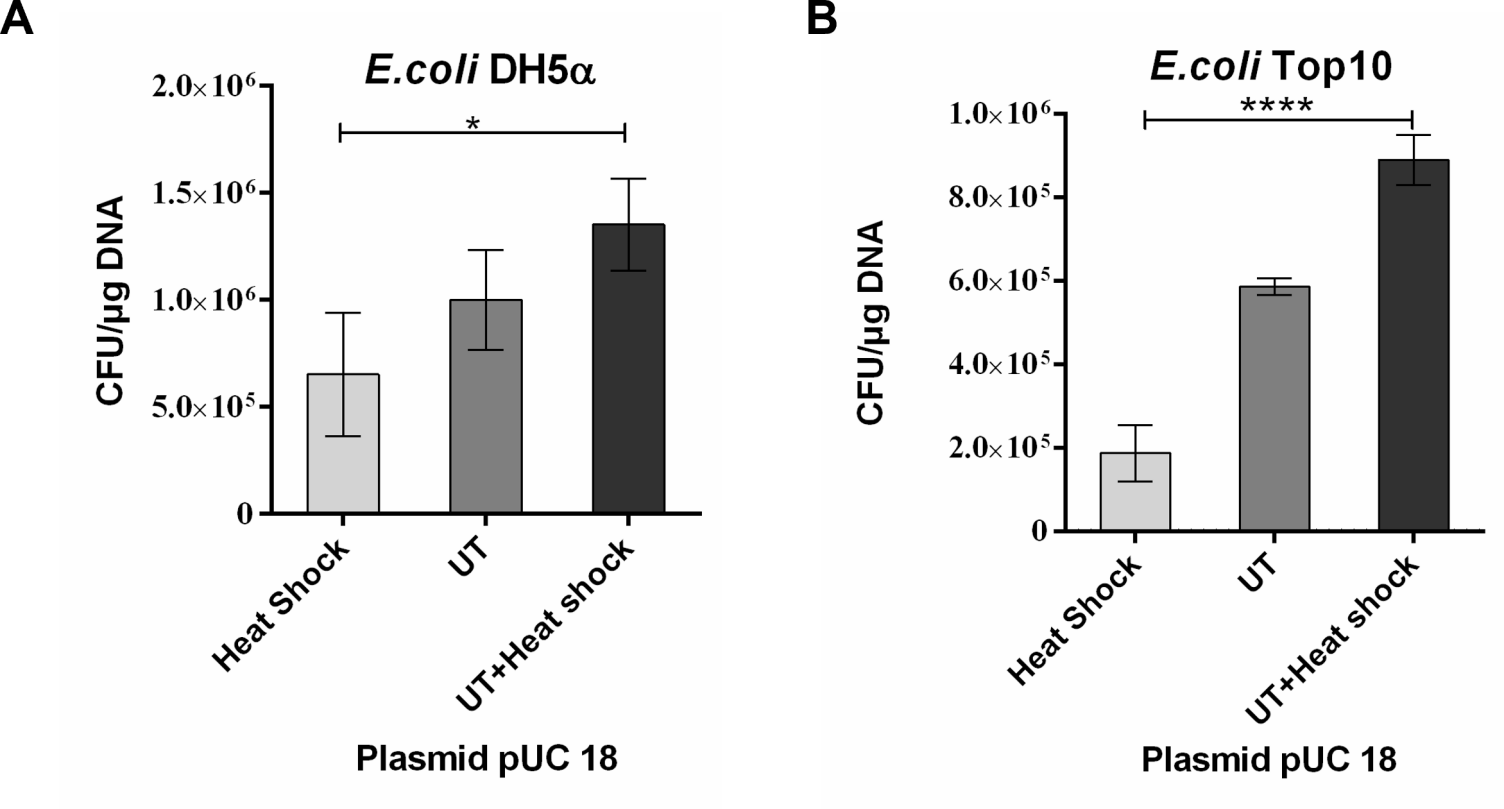
Ultrasonic treatment improved the plasmid transformation efficiency. (**A**) The host strain was DH5α. (**B**) The host strain was Top10. The *X*-axis represents the different transformation methods. The *Y*-axis represents the transformation efficiency. *0.01 < *P* < 0.05; **0.001 < *P* < 0.01; ***0.0001 < *P* < 0.001.

**Fig 3:**
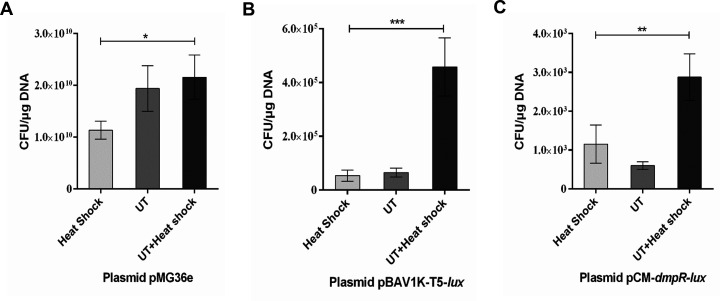
Ultrasonic treatment improved the plasmid transformation efficiency when the host strain was DH5α. The plasmids with different size were：(A) pMG36e, (B) pBAV1K-T5-lux, and (C) pCM-dmpR-lux. The *X*-axis represents the different transformation methods. The *Y*-axis represents the transformation efficiency. *0.01 < *P* < 0.05; **0.001 < *P* < 0.01; and ***0.0001 < *P* < 0.001.

Ultrasonic treatment based on heat shock transformation improved the transformation efficiency. Using two different competent cells, UT + Heat shock treatment exhibited the highest efficiency among the three transformation methods (Fig. 2). Considering three different plasmids, the Heat shock and UT + Heat shock groups differed significantly (Fig. 3). Transformation efficiency drops dramatically with increasing size of the DNA (38). The transformation system remained relatively static during the heat shock treatment, changed the membrane permeability with temperature, and promoted the entry of adsorbed plasmids into the cell. After ultrasonic treatment, the competent cell liquid in the transformation system was in a dynamic environment, promoting the movement of microbubbles. Cell contact was more conducive to the entry of plasmids into cells because ultrasonic treatment improved the efficiency of heat shock transformation.

### Influence of cryoprotective agents on ultrasonic transformation efficiency and toxicity

**Fig 4:**
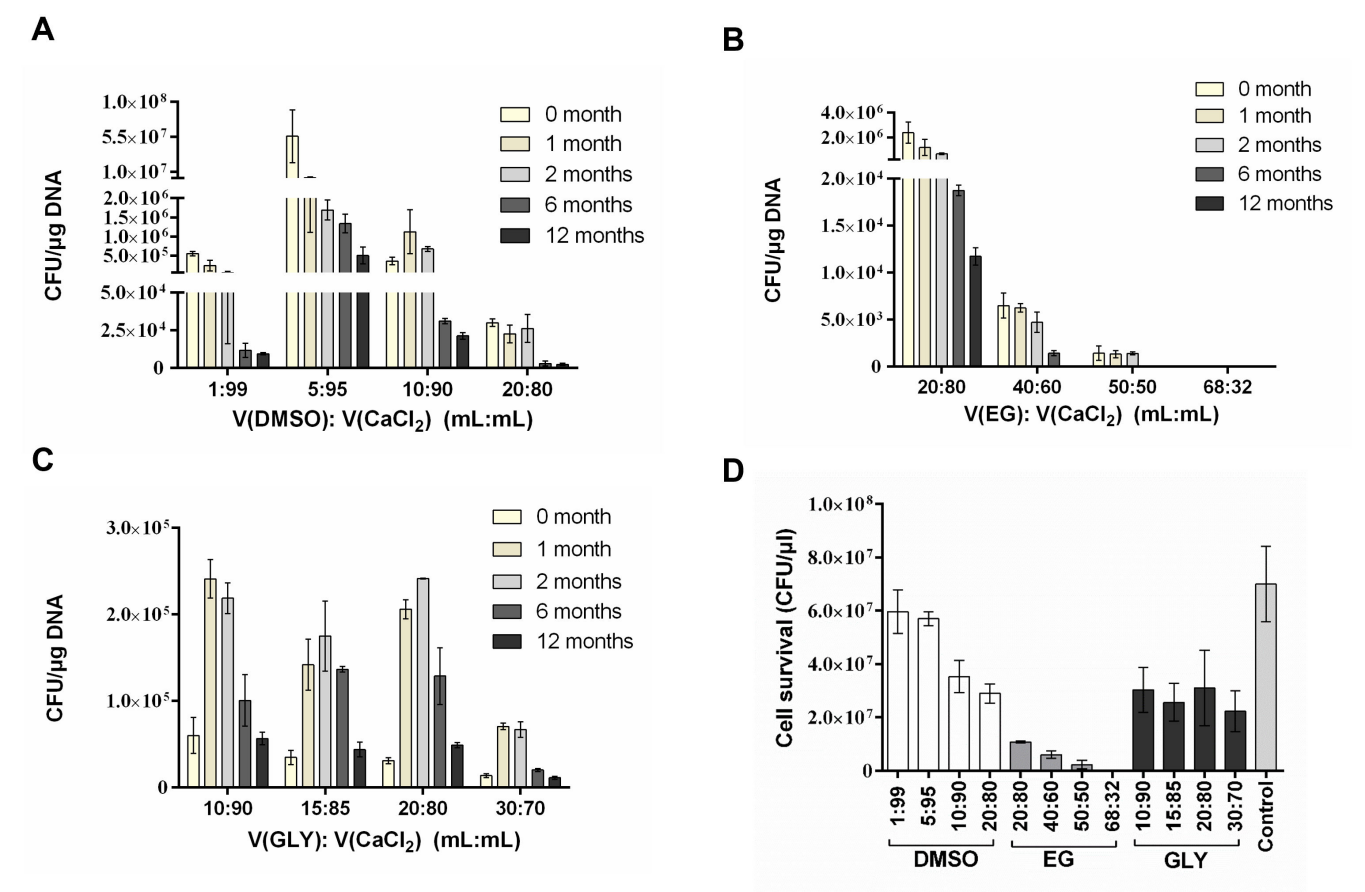
The influence of cryoprotective agents on ultrasonic transformation efficiency and toxicity. The host bacteria were DH5α, and the transformed plasmids were pUC18. (**A**) The cryoprotective agent prepared with DMSO and CaCl_2_ solution. (**B**) The cryoprotective agent prepared with ethylene glycol and CaCl_2_ solution. (**C**) The cryoprotective agent prepared with glycerol and CaCl_2_ solution. (**D**) The survival of competent cells treated with cryoprotective agent. The *X*-axis represents the different ratio of different cryoprotective agents. The *Y*-axis represents the cell survival (CFU/μL).

The composition of the CPAs for the DH5α competent cells was investigated to obtain the optimal cryopreservation effect and transformation efficiency. As shown in Fig. 4A, when the DMSO: CaCl_2_ solution (vol:vol) ratio was 5:95, the best ultrasonic transformation efficiency was obtained. The ultrasonic transformation efficiency reached 6 × 10^7^ CFU/µg DNA without cryopreservation. After 1 month of cryopreservation, the ultrasonic transformation efficiency reached 2 × 10^6^ CFU/µg DNA. The transformation efficiency gradually decreased with time. After cryopreservation for 12 months, the transformation efficiency still reached 5 × 10^5^ CFU/µg DNA, which was sufficient for normal transformation. In a previous study (24), an *E. coli* JM109 cryopreservation system used LB medium containing 10% (wt/vol) PEG, 5% (vol/vol) DMSO, and 20–50 mM Mg^2+^ (MgSO_4_ or MgCl_2_). When CPA contained Mg^2+^, adding 5% DMSO significantly enhanced the transformation efficiency. When 10% DMSO was used, the conversion level was reduced, and almost no conversion was observed with 25% DMSO. The trend of the results obtained in this study is similar to the data of our study.

When the EG: CaCl_2_ solution (vol:vol) = 20:80, the transformation efficiency was 1.2 × 10^6^ CFU/µg DNA after 1 month (Fig. 4B). With the increase of cryopreservation time, the transformation efficiency gradually decreased, and the transformation efficiency decreased to 1.1 × 10^4^ CFU/µg DNA after 12 months of storage. When GLY was used as an antifreeze, CPA with ratios of 10:90, 15:85, and 20:80 had similar preservation effects (Fig. 4C). After 2 months of cryopreservation, the ultrasonic transformation efficiency remained at approximately 2 × 10^5^ CFU/µg DNA. However, it was at least one order of magnitude different from that of the DMSO group.

This study verified the toxicity of different CPAs in DH5α competent cells. The number of viable competent cells was significantly reduced in each treatment group than in the control group (Fig. 4D). The number of competent cells in the control group was about 7 × 10^7^ CFU/µL. Among the 12 cryoprotectant treatment groups, the DMSO: CaCl_2_ solution (vol:vol) = 1:99 group had the largest number of surviving cells, the cell survival of the DMSO: CaCl_2_ solution (vol:vol) = 5:95 group was the second, and the cell survival could reach about 6 × 10^7^ CFU/µL. The EG: CaCl_2_ solution (vol:vol) = 68:32 group had the lowest cell survival, possibly leading to a lower ultrasonic transformation efficiency. The data were similar for the four solutions with different ratios in GLY groups, which remained stable at 2.2 × 10^7^–3 × 10^7^ CFU/µL. DMSO, EG, and GLY are cryoprotectants that cross cell membranes and interfere with ice crystal formation. Theoretically, toxicity is likely to occur after the penetration of the cryoprotectant into the cell. Excessive osmotic stress can interfere with protein structure and reduce enzyme activity while causing DNA damage and cell death.

In summary, this study optimized the transformation conditions for the ultrasound-mediated introduction of exogenous plasmids into DH5α. Moreover, this experiment combined traditional heat shock transformation with ultrasonic transformation. The experimental comparison verified that the transformation efficiency was highest using a 3-mL UT vial at 28 kHz ultrasound frequency, 42°C heat shock, and 80 W ultrasound power, providing a reference for further optimization of ultrasonic transformation conditions in *E. coli* DH5α. Meanwhile, this study optimized CPA (DMSO: CaCl_2_ solution [vol:vol] = 5:95) for competent cells and provided a new direction for further optimization of the transformation method.

## DISCUSSION

Briefly, the main purpose of this study was to improve the efficiency of ultrasound-mediated transformation and provide an option for efficient cryoprotective agents for competent cells. The technology of ultrasound-mediated transformation was initially applied to eukaryotic cells for gene therapy. It extended the application in the transformation of Gram-negative bacteria until 2007 (13). The ultrasound transformation efficiency is still lower than the conventional electrical method. However, differences in transformation efficiency can be affected by a variety of factors, the most obvious difference being the different batches of competent cells prepared.

In this study, the *E. coli* used was sub-cultured from commercially competent cells, and the strain is more suitable for transforming exogenous plasmids. Though the entire experimental procedure was authentic and reliable, it was not always feasible to achieve the highest efficiency in each batch of experiments. Hence, the efficiency of transformation was only compared in different groups within the same experiment. It was interesting to note that combining classical heat shock transformation with ultrasound-mediated transformation significantly increased the efficiency of transforming exogenous plasmids in the same experimental batch (Fig. 2).The results show that the optimized ultrasound-mediated transformation method is more suitable for the transformation of the plasmid pMG36e.

Overall, this study has optimized the existing sonication process and provided a more suitable cryopreservation solution for competent cells. It is worth discussing how to make the sonication method simpler or more applicable to the transformation of Gram-positive bacteria, which needs to be thoroughly researched in the future.

## SUPPLEMENTAL MATERIAL

Supplemental material spectrum.00978-24-s0001.docx Fig. S1 and S2.

ASM does not own the copyrights to Supplemental Material that may be linked to, or accessed through, an article. The authors have granted ASM a non-exclusive, world-wide license to publish the Supplemental Material files. Please contact the corresponding author directly for reuse.
