# Supplementary material for: Optimization of ultrasound-mediated DNA transfer for bacteria and preservation of frozen competent cells
Source: Microbiol Spectr. 2024 Nov 12;12(12):e00978-24. doi: 10.1128/spectrum.00978-24 (PMC11620496; doi:10.1128/spectrum.00978-24)
Supplement: Supplemental material — Fig. S1 and S2. [file spectrum.00978-24-s0001.docx]

**Supplementary Material**

**Title:** Optimization of ultrasound-mediated DNA transfer for bacteria and preservation of frozen competent cells

**

**

**Fig. S1** The pre-experiment result of ultrasonic power on the transformation efficiency.


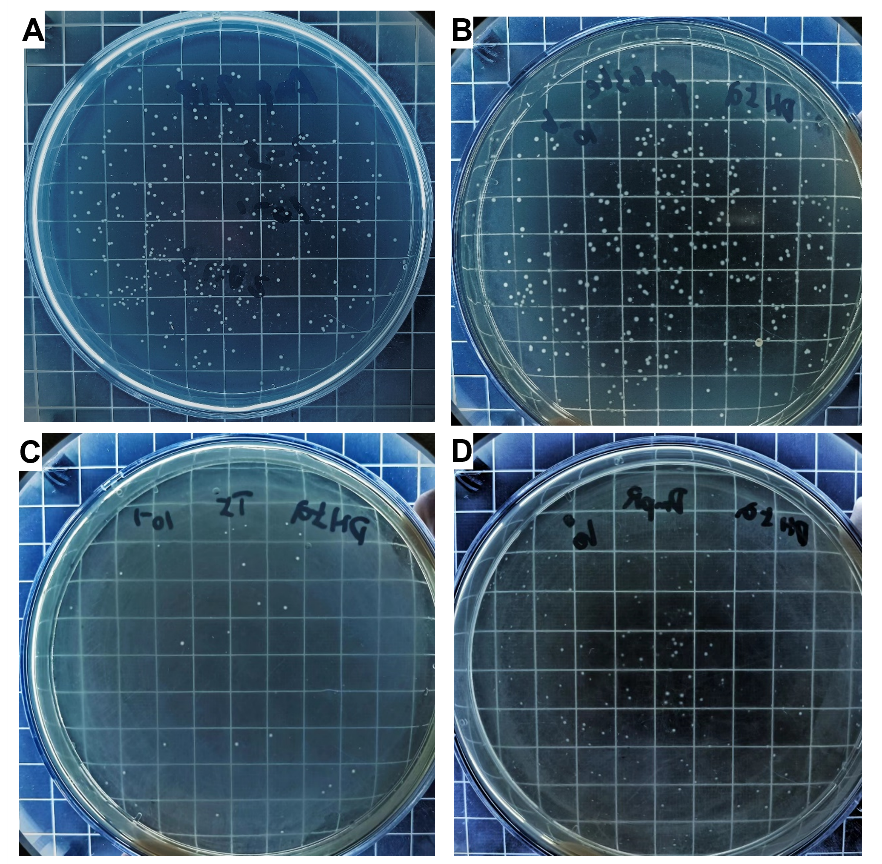


**Fig. S2** Four kinds of plasmids, pUC18(A), pMG36e (B), pBAV1K-T5-*lux*(C), pCM-*dmpR*-*lux*(D), were successfully transformed into DH5α by ultrasonic transformation.
